# A Case of Idiopathic Thrombocytopenic Purpura After Booster Dose of BNT162b2 (Pfizer-Biontech) COVID-19 Vaccine

**DOI:** 10.7759/cureus.18985

**Published:** 2021-10-23

**Authors:** Srikrishna V Malayala, Bhavani N Papudesi, Rishika Sharma, Urwat T Vusqa, Ambreen Raza

**Affiliations:** 1 Internal Medicine, Temple University Hospital, Philadelphia, USA; 2 Internal Medicine, Mercy Health, Philadelphia, USA; 3 Pediatric Medicine, Kaweah Delta Health Care District, Visalia, USA; 4 Internal Medicine, Allegheny Health Network, Pittsburgh, USA; 5 Internal Medicine, Bayhealth Hospital, Dover, USA

**Keywords:** booster side effects, vaccine side effects, covid-19 vaccine complication, mrna-based vaccine, idiopathic thrombocytopenic purpura, covid-19 vaccine, drug-induced itp, covid 19, pfizer-biontech covid-19 vaccine, covid associated thrombocytopenia

## Abstract

Vaccination is now considered the best measure in minimizing the morbidity and mortality from the Covid-19 pandemic. Almost all the vaccines are considered safe except for minor and occasional side effects. Some of the commonly reported complications from the COVID-19 vaccines are vaccine-induced thrombotic thrombocytopenia (VITT)/thrombosis with thrombocytopenia syndrome/vaccine-induced pro-thrombotic immune thrombocytopenia syndrome.

In this case report, we present a case of a 75-year-old female who had an uncomplicated first and second vaccine dose but developed VITT after the booster dose of the vaccine. The patient was treated with dexamethasone and platelet transfusions. So far no such cases have been reported after the third (booster) dose of the Pfizer-Biontech vaccine.

With this case report, we present the case of the patient and discuss the literature related to vaccine-induced thrombocytopenia.

## Introduction

As an immediate relief to the raging COVID-19 pandemic in the United States (US) and all across the globe, the US Food and Drug Administration (FDA) issued the emergency use authorization of the Pfizer-BioNTech COVID-19 vaccine (BNT162b2) in December 2020 [1]. It was initially recommended for all individuals aged 16 years of age and older and later expanded to anyone more than 12 years old [2].

Ever since its introduction and administration, the side effect profile of the vaccine is being closely monitored. The common side effects reported post-vaccine are diarrhea, nausea and vomiting, local pain at the injection site, swelling at the injection site, chills, fatigue, headache, myalgias, and arthralgias [3,4]. A few cases of thrombocytopenia have been reported as a side effect of the administration of COVID-19 vaccines. As of June 2021, about 21 cases of thrombocytopenia following vaccination were reported out of which 17 were without any pre-existing evidence of thrombocytopenia. About 14 cases were had bleeding episodes associated with thrombocytopenia [5,6].

The recommended dose of the BNT162b2 vaccine is two shots, three weeks apart from each other. It is now recommended that moderate to severely immunocompromised individuals should get a booster (third) dose four weeks after the second dose. Center for Disease Control, CDC defines this set as people receiving chemotherapy, organ transplant recipients, untreated HIV infection, post stem transplant, and active treatment with glucocorticoids [7]. Everyone else should receive the booster dose six months after their second dose [7].

In this case report, we present a patient who developed immune thrombocytopenic purpura (ITP) after receiving the booster dose of the Pfizer Covid-19 vaccine. The patient in our case report has a history of scleroderma and rheumatoid arthritis and was being treated with methotrexate and hydroxychloroquine. She received the booster dose of the vaccine and within four days, she presented to the emergency room with thrombocytopenia and purpura. 

## Case presentation

A 75-year-old female patient presented to the emergency room for thrombocytopenia found on routine bloodwork ordered by her primary care physician. At the baseline, she was never diagnosed with thrombocytopenia or any other platelet disorder. The patient was found to have a low platelet count of 75,000/mm^3^ which fell further down to 18,000/mm^3^ in a span of five days at which point the patient was sent to the emergency room for further evaluation. The patient denied any overt bleeding but reported noticing a small left lower extremity and a few petechiae five days before arrival to the emergency room. The patient denied fever/chills/nausea or vomiting.

The patient has a known history of rheumatoid arthritis, scleroderma, mixed connective tissue disease, hypertension, cardiac disease, and osteopenia. The patient has been taking methotrexate and hydroxychloroquine for many years and tolerated them well without any adverse effects. She takes 1 mg oral folic acid three times a week. Medication history also includes dual anti-platelet therapy with aspirin and clopidogrel. She stopped taking methotrexate two weeks before presenting to the hospital as advised by her rheumatologist as she was going to receive her third COVID-19 vaccine. The patient received her third dose of Pfizer COVID-19 vaccine four days before the lab work that showed low platelet count for the first time. The patient's platelet count was 198,000/mm^3^ one week prior to the vaccine (Table 1). 

**Table 1:** Platelet counts of the patient through the hospital course

Hospital day course	Platelet count (K/mm^3^)
One week prior to admission	198
Day of admission	18
In the emergency room	9
Post transfusion	24
Day 2	21
Day 3	19
Day 4	25
Day 5	53
Day 6	61

In the emergency room, she was found to be hemodynamically stable with laboratory data showing a low platelet count of 9000/mm^3^ (normal reference range 150,000-400,000/mm^3^), hemoglobin of 12 g/dl (normal reference range 12-18 g/dl) with a normal white cell count of 5900/µl (normal reference range 5000-11,000/mm^3^). The international normalized ratio (INR) was reported as 1.1 (normal range 08-1.2). Protime (PT) was 13.9 seconds (normal reference range 10-14 seconds) and activated partial thromboplastin clotting time (aPTT) was 32.3 seconds (normal reference range 25-36 seconds). The d-dimer level was marginally elevated to 1.22 µg/ml (normal value is <0.44 µg/ml). Vitamin B12 and folic acid levels and hepatic function levels were within normal limits. Ultrasound abdomen revealed hepatic steatosis but spleen was normal in size. The peripheral smear did not reveal abnormalities (Table 2).

**Table 2:** Patient's laboratory range at admission

Laboratory parameter	Admission value	Reference range
Hemoglobin	12 g/dl	12–18 g/dl
WBC	5900/µl	5000–11,000/mm^3^
Platelet count	9000/mm^3^	150,000–400,000/mm^3^
d-dimer	1.22 µg/ml	<0.44 µg/ml
INR	1.1	0.8-1.2
Prothrombin time	13.9 seconds	10–14 seconds
Activated partial thromboplastin clotting time	32.3 seconds	25–36 seconds

There were no factors that could cause the thrombocytopenia and she was admitted with the chief diagnosis of idiopathic thrombocytopenic purpura (ITP). On admission, aspirin and clopidogrel were discontinued. The patient was started on dexamethasone 40 mg by mouth daily. She was given a unit of platelet transfusion. Post transfusion, platelet level increased to 24,000/mm^3^. The systemic steroids were continued and platelet counts were monitored. She went into a transient phase of steroid-induced delirium that resolved once the proposed five-day course of dexamethasone was completed. Her platelet counts gradually improved. 

With no other triggering factor and based on the available literature, a diagnosis of ITP induced by the booster dose of COVID-19 Pfizer vaccine (BNT162b2) was made. The patient was discharged home with outpatient follow-up. Aspirin was restarted due to the patient's history of coronary artery disease but clopidogrel was held as the bleeding risk was still considered high.

## Discussion

We report a patient with ITP after the booster (third) dose of Pfizer-BioNTech COVID-19 vaccine, BNT162b2. The signs and symptoms of thrombocytopenia occurred immediately after the vaccination and they could not be explained by any other etiology, thus confirming the diagnosis of vaccine-induced ITP. There were no other clinical signs or symptoms or laboratory data to explain other potential differential diagnoses like thrombotic thrombocytopenic purpura, hemolytic uremic syndrome, disseminated intravascular coagulation, or drug-induced thrombocytopenia. The patient was tolerating aspirin and clopidogrel without any incidence of thrombocytopenia.

The pathophysiology of the vaccine-induced ITP is a complicated phenomenon. The components used to develop the commonly used vaccines like ovalbumin, gelatin, and certain milk proteins have been held responsible for acute thrombocytopenia after the vaccine administration [8]. However, these components are absent in both the Pfizer-BioNTech and Moderna COVID-19 vaccines [8]. The mRNA component of the vaccine is unlikely to instigate an allergic reaction by itself but the modified RNA trace impurities that are present in the Pfizer-BioNTech COVID-19 vaccine could explain the thrombocytopenia [9]. There is a possibility that these aberrant proteins could initiate an immunological reaction that can lead to thrombocytopenia [9].

The COVID-19 mRNA-based vaccines are new vaccines that are different from the traditional inactivated or live-attenuated vaccines. These vaccines contain RNA that encodes the spike protein encapsulated within a lipid nanoparticle (LNP) [10]. The side effects related to COVID-19 vaccines are possibly associated with LNPs. They are reported in the Vaccine Adverse Events Reporting System (VAERS) [10]. In the patient presented in our case, the LNPs could have induced an immune reaction against platelets, resulting in vaccine-induced ITP.

The patient in our case responded to dexamethasone and had a steady platelet level thereafter. Very limited information is available to suggest that IVIG and high dose steroids as initial treatment. If there is no response to systemic steroids, any of the thrombopoietic agents could be tried as well [11]. The commonly used rituximab from initial treatment is not recommended in most cases given that the response to vaccination can be impaired [12].

With the vaccine being less than one year old, it is not known if the ITP in these cases could be self-limiting or lead to a chronic ITP [13]. Thrombocytopenia reports after COVID-19 vaccines do not exceed the background rate of ITP [14,15]. The reporting rate of thrombocytopenia is 0.8 per million doses [8]. The number of thrombocytopenia cases reported to VAERS does not suggest a safety concern attributable to mRNA COVID-19 vaccines at this time [10].

Vaccine-induced thrombotic thrombocytopenia (VITT) is a similar condition that is reported as a side effect of the Johnson & Johnson/Janssen and AstraZeneca COVID-19 vaccines. However, this condition has not been reported in patients who received the commonly used mRNA vaccines like Moderna or Pfizer vaccines. Unlike VITT, the risk of thrombosis with ITP is usually not increased, and anticoagulation, which is a suggested treatment in VITT, is not considered [16].

## Conclusions

In the current era, the m-RNA and other vaccines are the most effective weapon against the deadly COVID-19 pandemic. Clinicians should be aware of the presenting features of vaccine-induced thrombocytopenia. A high suspicion should arise when a patient presents with clinical features of thrombocytopenia after the covid vaccine irrespective of the number of doses or brand of the vaccine. As in this case, it is evident that patients who did not have any symptoms with the first and second dose could develop thrombocytopenia after the third dose. With the unpredictable number of mutant variants that might emerge, there is a high possibility that multiple booster doses could be required in the future. There is a potential to develop ITP with any of those boosters despite tolerating the regular doses or prior booster doses without any complications. A delay in diagnosis and treatment could lead to catastrophic bleeding complications.

The purpose of this article is only to increase the awareness of possible complications that will lead to early detection and treatment and not to diminish the safety standards of the Pfizer-BioNTech COVID-19 vaccine.
